# *In Vivo* and *In Silico* Studies of the Hepatoprotective Activity of *Tert*-Butylhydroquinone

**DOI:** 10.3390/ijms25010475

**Published:** 2023-12-29

**Authors:** Liseth Rubi Aldaba-Muruato, Sandra Sánchez-Barbosa, Víctor Hugo Rodríguez-Purata, Georgina Cabrera-Cruz, Estefany Rosales-Domínguez, Daniela Martínez-Valentín, Yoshio Aldo Alarcón-López, Pablo Aguirre-Vidal, Manuel Alejandro Hernández-Serda, Luis Alfonso Cárdenas-Granados, Víctor Hugo Vázquez-Valadez, Enrique Angeles, José Roberto Macías-Pérez

**Affiliations:** 1 Biomedical Science Laboratory, Clinical Chemistry, Faculty of Professional Studies Huasteca Zone, Autonomous University of San Luis Potosi, Ciudad Valles 79060, San Luis Potosi, Mexico; liseth.aldaba@uaslp.mx (L.R.A.-M.); profra.sandrasb@gmail.com (S.S.-B.); a291170@alumnos.uaslp.mx (G.C.-C.); a265171@alumnos.uaslp.mx (E.R.-D.); a275542@alumnos.uaslp.mx (D.M.-V.); 2 Pharmacobiological Sciences, Faculty of Chemical Sciences, Autonomous University of San Luis Potosi, San Luis Potosi 78210, Mexico; a283438@alumnos.uaslp.mx; 3 Laboratorio de Química Teórica y Medicinal, FESC, Universidad Nacional Autónoma de México, Avenida 1 de Mayo S/N, Santa María las Torre, Cuautitlán Izcalli 54750, Estado de México, Mexico; yoshalar@gmail.com (Y.A.A.-L.); pyogenes2heli@gmail.com (P.A.-V.); serda@cuautitlan.unam.mx (M.A.H.-S.); aalfonsocardenas@gmail.com (L.A.C.-G.); hugounam83@gmail.com (V.H.V.-V.); angeles@unam.mx (E.A.)

**Keywords:** acute liver damage, cholestasis, necrosis, mortality, survival, in silico studies

## Abstract

*Tert*-butylhydroquinone (TBHQ) is a synthetic food antioxidant with biological activities, but little is known about its pharmacological benefits in liver disease. Therefore, this work aimed to evaluate TBHQ during acute liver damage induced by CCl_4_ (24 h) or BDL (48 h) in Wistar rats. It was found that pretreatment with TBHQ prevents 50% of mortality induced by a lethal dose of CCl_4_ (4 g/kg, i.p.), and 80% of BDL+TBHQ rats survived, while only 50% of the BDL group survived. Serum markers of liver damage and macroscopic and microscopic (H&E staining) observations suggest that TBHQ protects from both hepatocellular necrosis caused by the sublethal dose of CCl_4_ (1.6 g/kg, i.p.), as well as necrosis/ductal proliferation caused by BDL. Additionally, online databases identified 49 potential protein targets for TBHQ. Finally, a biological target candidate (Keap1) was evaluated in a proof-of-concept in silico molecular docking assay, resulting in an interaction energy of −5.5491 kcal/mol, which was higher than RA839 and lower than monoethyl fumarate (compounds known to bind to Keap1). These findings suggest that TBHQ increases the survival of animals subjected to CCl_4_ intoxication or BDL, presumably by reducing hepatocellular damage, probably due to the interaction of TBHQ with Keap1.

## 1. Introduction

Worldwide, there are approximately two million deaths caused by liver disease, of which half are from cirrhosis and the other half from viral hepatitis and hepatocarcinoma [1]. The liver is a multifunctional organ susceptible to damage due to several factors, such as the consumption of alcohol or drugs, obstruction of bile flow, exposure to toxins, or parasitic and viral infections [2,3,4]. Certainly, regardless of the causative agent of liver damage, hepatocyte cell death favors the progression of the disease, starting with inflammation that leads to fibrosis and finally to liver cancer [3,5]. However, despite medical advances, there are no effective treatments that can prevent or even reverse liver disease, with organ transplantation being the procedure of choice for irreversible liver damage. However, there is a growing number of patients waiting for a liver transplant that exceed the number of donors [6].

Liver pathogenesis manifests itself as inflammation and necrosis, cholestasis, fibrosis, cirrhosis, and hepatocarcinoma [1,7]. In the present investigation, the hepatoprotective capacity of *Tert*-butylhydroquinone (TBHQ) was evaluated during acute liver damage induced by extrahepatic cholestasis and carbon tetrachloride (CCl_4_) intoxication, and its possible mechanism of protection was hypothesized by bioinformatic and in silico analysis.

**Figure 1:**
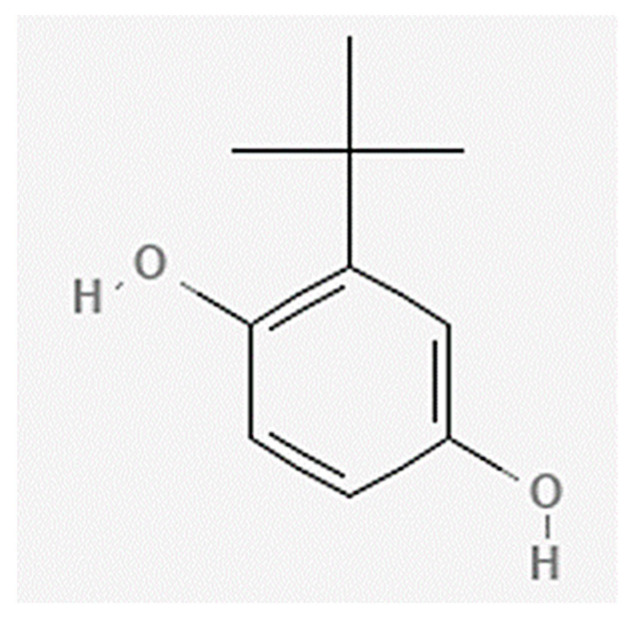
Chemical structure of TBHQ.

TBHQ (CH3)3CC6H3-1,4-(OH)2 is a crystalline powder, a member of the class of hydroquinones in which one of the hydrogens in the hydroquinone ring is replaced by a tert-butyl group (Figure 1). This compound is commonly used as a synthetic food antioxidant to prevent oxidative deterioration and the rancidity of oils and fats due to its potent activity to inhibit lipid peroxidation [8,9]. Recent studies attribute various biological activities, such as the preservation of testicular steroidogenesis and spermatogenesis, to improving ethanol-induced gastric ulcers and radiocontrast-induced nephropathy [10,11,12].

**Figure 2:**
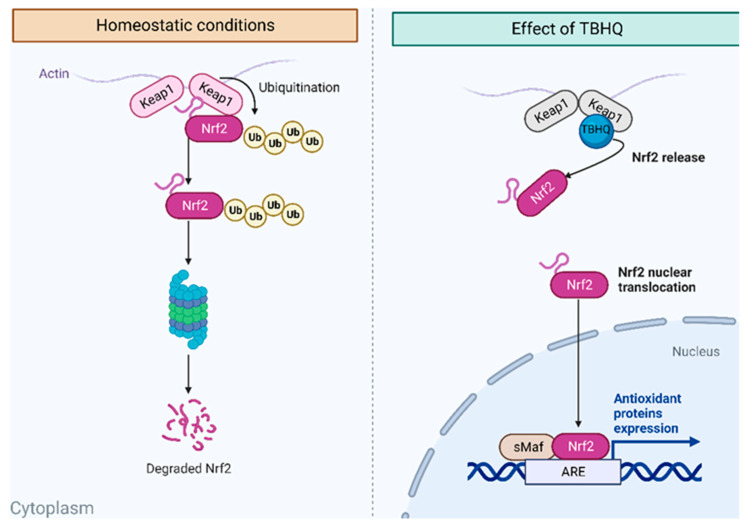
Homeostatic conditions of Nrf2–Keap1 and the proposed effect of TBHQ on the Nrf2–Keap1 pathway. Image created from BioRender.com.

The bioactive effect of TBHQ is attributed to the induction of phase II drug-metabolizing enzymes through a dependent pathway of nuclear factor erythroid 2-related factor 2 (Nrf2), which is a member of the basic leucine zipper transcription factor (bZIP), regulated mainly by Kelch-like ECH-associated protein 1 (Keap1) [13]. Keap1 is an adapter substrate of the Cullin–RING E3 ubiquitin ligase complex, which suppresses Nrf2 in the cytoplasm by sequestration, ubiquitination, and proteasomal degradation [14]. Under the stimulus of reactive oxygen species (ROS) or electrophilic agents, the hyperreactive cysteine residues of Keap1 allow the inactivation of the E3 ubiquitin ligase, and Nrf2 is dissociated from Keap1. Nrf2 accumulates in the cytoplasm and travels to the nucleus to mediate cytoprotective gene expression through antioxidant-responsive elements (AREs). The Keap1 protein contains a domain called BTB (bric-a-brac) that mediates dimerization, another domain called the IVR (intermediate region) domain that is involved with the interaction with CUL3, and a joint domain called DC composed of the individual domains DGR (double glycine repeat) and CTR (carboxyl-terminal region). In this DC domain, the interaction with Nrf2 occurs. On the Nrf2 protein, there are DLG and ETGE motifs in the section called Neh2 (amino end) that interact with the DC domains of the Keap1 homodimer [15,16]. Therefore, another objective of the present study was to analyze the interaction between TBHQ and Keap1 in silico (Figure 2).

## 2. Results

### 2.1. Mortality and Survival

The CCl_4_ model allowed us to evaluate the protective capacity of TBHQ against lethal and sublethal toxicity induced by CCl_4_ (Table 1). After 24 h, a lethal dose of CCl_4_ (4 g/kg, i.p.) yielded 100% (6/6) mortality, while TBHQ pretreatment prevented 50% (3/6) of mortality induced by the lethal dose. However, all animals injected with a sublethal dose of CCl_4_ (1.6 g/kg, i.p.) survived. In addition, bile duct ligation (BDL group, 48 h) leads to 50% mortality and pretreatment with TBHQ shows a mortality of 20% (80% survival).

### 2.2. Body and Liver Weights

Statistical analysis of body weights, liver weights, and liver/body ratio was performed in both experimental models of liver damage (Table 2). Body weight was significantly reduced in the CCl_4_+TBHQ group in relation to the CCl_4_ and TBHQ groups, while liver weight increased in the CCl_4_ and CCl_4_+TBHQ groups compared to the Control group. Furthermore, liver/body weight increased significantly in the CCl_4_ and CCl_4_+TBHQ groups compared to the TBHQ and Control groups. However, the SHAM (simulated surgery), BDL, BDL+TBHQ, and TBHQ groups did not show statistically significant differences between them for any of the weights.

### 2.3. Macroscopic Findings of Livers

**Figure 3:**
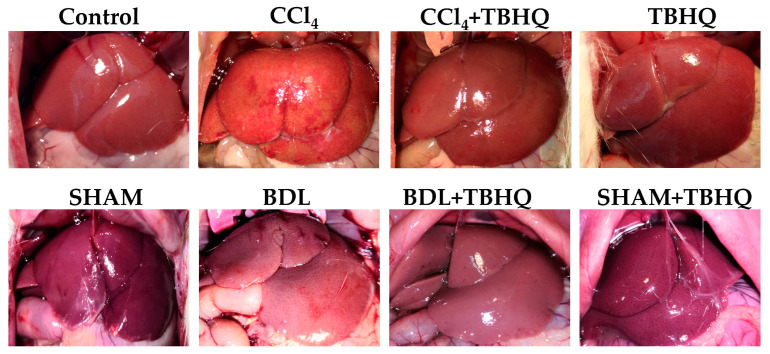
Representative images of in situ livers of rats from the CCl_4_ and BDL models.

Livers were observed in situ (Figure 3), and healthy control groups (Control, TBHQ, SHAM, and SHAM+TBHQ) presented a uniform and smooth surface with soft consistency on palpation. Additionally, livers in the BDL group showed an altered appearance with a pale surface, while the CCl_4_ group presented an altered hepatic morphology with a necrotic appearance. The gross appearance of the livers from the CCl_4_+TBHQ and BDL+TBHQ groups showed a less damaged appearance compared to the damage groups (CCl_4_ or BDL, respectively).

### 2.4. Microscopic Evaluation of Liver Damage Induced by a Sublethal Dose of CCl_4_

**Figure 4:**
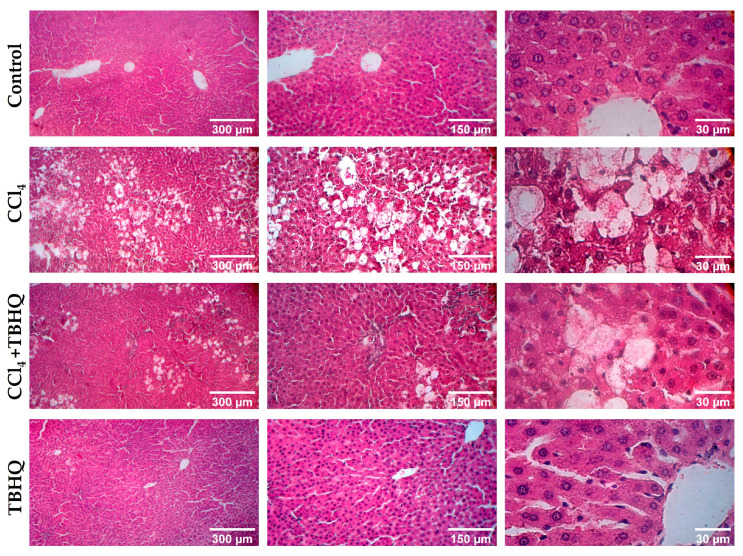
Representative micrographs of H&E-stained liver tissue sections from the CCl_4_ model. Photographs obtained from samples at different magnifications (5X, 10X, and 40X) show the differences and alterations in the tissue according to the different treatment groups.

The hepatoprotective activity of TBHQ was evaluated during the sublethal dose of CCl_4_ with hematoxylin–eosin (H&E) staining (Figure 4). The hepatic parenchyma of the Control and TBHQ groups showed a normal architecture characteristic of healthy livers, with normal organization of hepatocytes organized in cords, in addition to clearly identifying some central veins and hepatic sinusoids, as shown in the micrographs. On the other hand, micrographs of the CCl_4_ group showed severe damage with evident steatosis and inflammatory infiltrate with ballooned hepatocytes; however, in liver samples of the CCl_4_+TBHQ group, less steatosis and ballooning degeneration were observed, showing better architectural integrity than the CCl_4_ group, whose damage was greater.

### 2.5. Microscopic Evaluation of Liver Damage Induced by BDL

**Figure 5:**
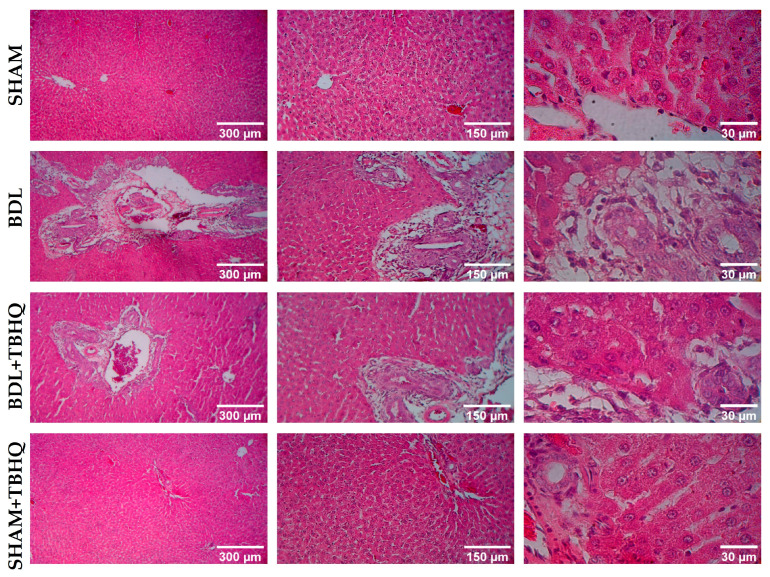
Representative micrographs of liver sections stained with H&E from the BDL model. Photographs obtained from samples at different magnifications (5X, 10X, and 40X).

H&E staining showed that two days after surgery, the BDL group presented liver necrosis, with a loss of normal architecture of the liver cords and infiltration of inflammatory cells with marked proliferation of the bile ducts. However, treatment with TBHQ prevented acute liver damage induced by BDL, since the BDL+TBHQ group showed little infiltration of inflammatory cells and mild proliferation of the bile ducts, as well as less hepatic necrosis and preservation of cell integrity, which can be compared to that of the healthy groups (Figure 5).

### 2.6. Serum Biochemical Markers of Liver Damage

**Figure 6:**
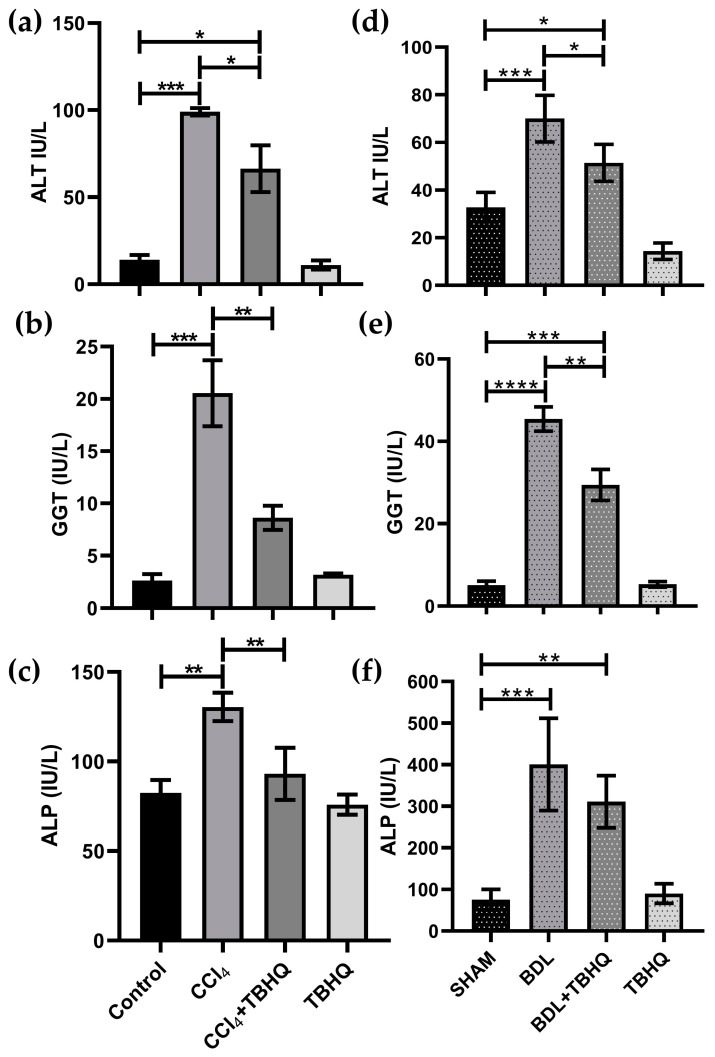
Evaluation of serum markers for liver damage. (**a**,**d**): ALT; (**b**,**e**): GGT; (**c**,**f**): ALP. CCl_4_ model (**a**–**c**): Control, CCl_4_: CCl_4_ (1.6 g/kg, i.p.), CCl_4_+TBHQ: CCl_4_ (1.6 g/kg, i.p.) + TBHQ pretreatment and TBHQ groups BDL model; (**d**–**f**): SHAM: SHAM surgery; BDL: bile duct ligation; BDL+TBHQ and SHAM+TBHQ groups. Results were expressed as the mean ± SE. Statistically significant differences: (*) *p* < 0.05, (**) *p* < 0.01, (***), *p* < 0.001, and (****) *p* < 0.0001.

Hepatic functionality was evaluated using the serum enzymatic activities of alanine aminotransferase (ALT), gamma-glutamyl transpeptidase (GGT), and alkaline phosphatase (ALP) (Figure 6). Intoxicated animals with a sublethal dose of CCl_4_ (Figure 6a–c) or with biliary obstruction (Figure 6d–f) showed significant increases in ALT, ALP, and GGT, respectively. Compared to the CCl_4_ group, TBHQ treatment (CCl_4_+TBHQ) partially but significantly prevented ALT (Figure 6a) and completely prevented increases in GGT (Figure 6b) and ALP (Figure 6c), similarly, to the Control group. Similarly, BDL and TBHQ partially prevent the increase in enzymatic activity of ALT, GGT, and ALP. The healthy groups (Control, TBHQ, SHAM, and SHAM+TBHQ) did not show significant differences between them in the enzymatic markers evaluated.

### 2.7. Identification of Potential TBHQ Protein Targets in the Liver

The possible mechanism of action of TBHQ was hypothesized using online platforms to search for its potential protein targets and data curated from the public Human Protein Atlas database. Thus, 49 potential targets for the THBQ protein were chosen (Table 3) if readily indicated as liver, expressed in liver, enriched in hepatocytes, liver stellate cells, or Kupffer cells (liver-specific macrophages), or were subunits of nuclear factor-kappa B (NF-κB), which is known to be involved in liver damage [17].

### 2.8. Molecular Docking of TBHQ and Keap1

**Figure 7:**
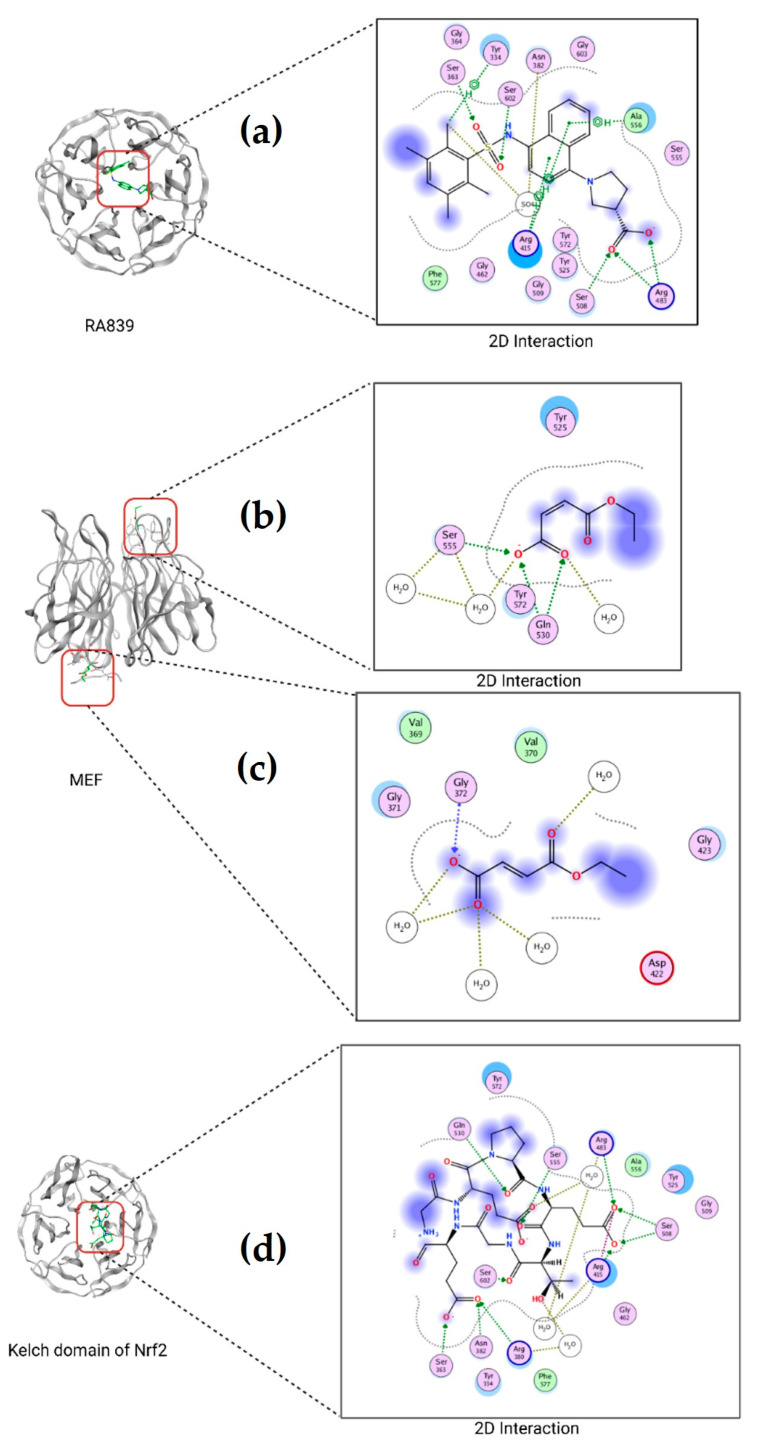
Three-dimensional and two-dimensional interaction diagrams. Keap1 with (**a**) RA839, (**b**,**c**) MEF, and (**d**) the Kelch domain of Nrf2.

The molecular systems found in the PDB files mentioned above showed that the amino acids in Keap1 interact with the residues Arg415, Ala556, Arg483, Arg483, Tyr334, Ser363, and Ser602 of (3S)-1-[4-[(2,3,5,6-tetramethylphenyl) sulfonylamino] -1-naphthyl] pyrrolidine-3-carboxylic acid, named as RA839 (Figure 7a). Examination showed that the amino acids in Keap1 that interact with monoethyl fumarate (MEF) are present in two regions: the first consisted near the residues Gln530, Tyr525, and Tyr572 (Figure 7b) and near the residues Asp422, Gly423 and Gly371, Gly372, Val369, and Val370 (Figure 7c). The information obtained from the Keap1 complex of Keap1 and the N-terminal region of Nrf2 showed that the Keap1 residues involved in the interaction are Arg415, Arg483, Ser508, Tyr525, Gln530, Ser555, Ala556, Tyr572, and Phe577 (Figure 7d).

**Figure 8:**
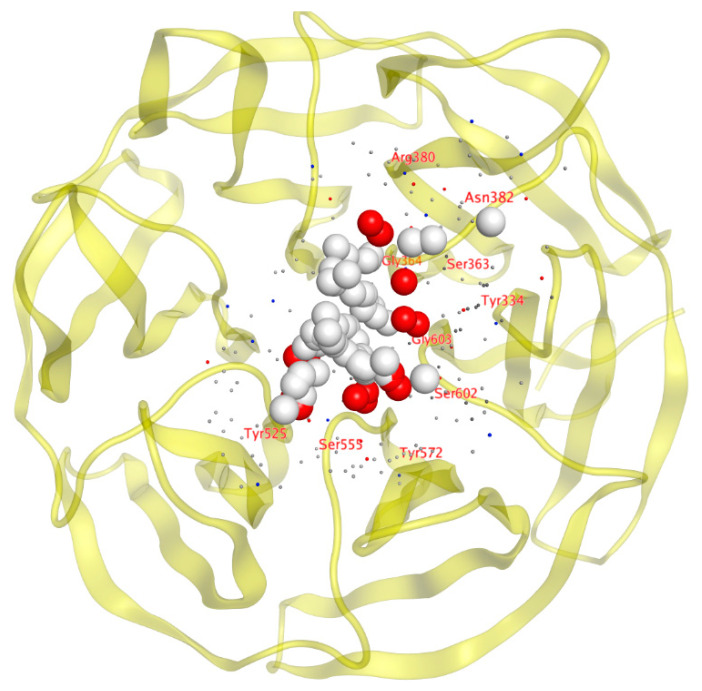
Common residues involved in the interaction present in each complex between the molecule and Nrf2. The MOE site-finding tool provides a high probability region filled with a set of dummy atoms for further docking experiments. Among the near residues are Arg380, Asp382, Ser363, Gly364, Tyr334, Gly603, Ser602, Tyr572, Ser555, and Tyr525.

Based on the previously described examination, we identified the common residues in the interaction present in each complex between the molecule and Nrf2. In the case of RA839, the Keap1 amino acids Arg 415, Arg483, and Ala556 are also involved in the interaction between Keap1 and Nrf2. For MEF, the Keap1 amino acids Gln530, Tyr525, and Tyr572 are also involved in the interaction of Keap1 with Nrf2. Using SiteFinder, a potential binding site was found near the residues shown in Table 4 and Figure 8, highlighting the common residues involved in the interaction of the PDB files.

During the docking experiments, the three cocrystallized ligands were re-evaluated to assess their energetic binding affinity. The interaction energy between the ligand obtained from PDB 7K2M, corresponding to the Nef2 domain of Nrf2, was observed to be the highest of all. The interaction of the MEF (from PDB 7C60) and RA839 (from PDB 5CGJ) molecules has lower interaction energies. When comparing the resulting interaction energy for TBHQ, it was higher than that of MEF but lower than that of RA839 (Table 5).

**Figure 9:**
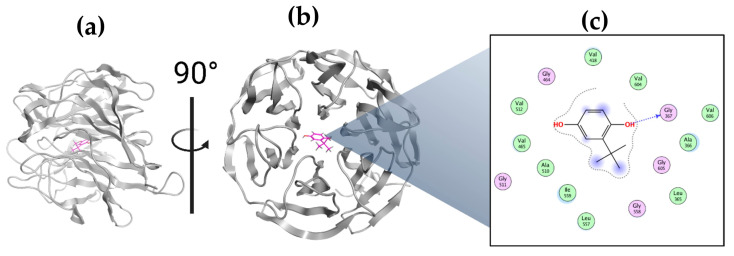
Three-dimensional and two-dimensional interaction diagrams of Keap1 with TBHQ: (**a**) front, (**b**) side, and (**c**) 2D.

Here, we examined the modeled interaction between Keap1 and TBHQ. At the modeled site, the ligand is deeply incorporated into the channel that Keap1 forms. This explains the greater binding affinity of the ligand‒receptor complex. If we look inside the 2D interactions, we can see that the contour (dotted line in Figure 9) covers most of the molecular surface of TBHQ and that the ligand exposure (marked with faded purple circles) is small. The strongest interaction is due to the hydrogen bonding of one hydroxyl group with Gly-367. The rest of the interactions responsible for stabilizing the complex are due to electrostatic interactions, protein surface complementarity, and contact preferences. The nearest residues are Gly367, Val604, Gly605, Gly464, Gly 558, and Val418, among others.

**Figure 10:**
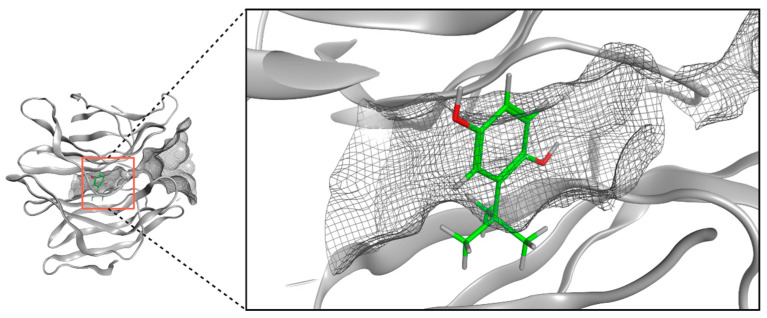
Three-dimensional interaction diagram of Keap1 with TBHQ allocated deep in the channel.

Although the site of interaction of this simulated complex is far from the X-ray structures that we found and described above, the size of the molecule could be an explanation for the position of the ligand in the protein. Considering that a channel is formed in the structure of Keap1 and due to the size of TBHQ, after replication of the simulation, TBHQ was always allocated deep into the channel, maintaining good binding energies (Figure 10).

## 3. Discussion

Approximately 2 million people die from liver disease each year due to the poor effectiveness of therapeutic treatments, leading to increased morbidity and mortality rates around the world [1]. Therefore, it is necessary to search for new compounds that can mitigate, prevent, or reverse liver damage. In the present work, we evaluated TBHQ as a possible hepatoprotective agent due to its anti-inflammatory, anti-apoptotic, and antioxidant properties that have been previously described [18,19,20]. Similarly, various in vitro and in vivo studies have demonstrated that TBHQ exhibits chemopreventive effects, although this compound has also been described as carcinogenic, though mainly when used at high concentrations [13]. In the CCl_4_ model used in the present work, two doses of TBHQ were used: 40 mg/kg, i.p., and 16.7 mg/kg, i.p., initially, TBHQ was administered for two days at 40 mg/kg, i.p., daily [21,22]; this dose in in vivo models increases the long-term stability of curcumin [21] and leads to induction of UGT1A1, through the Nrf2–Keap1 pathway [22]. However, on the second day, immediately after administration, animals became lethargic for 5 to 10 min and recovered (at this dose and the route of administration in experimental animals, we were unable to find these side effects reported in the literature). Taking into account these side effects, the dose was reduced to 16.7 mg/kg, i.p., every 8 h [19], and no adverse effects were observed in any of the animals. This dose of TBHQ reduced ischemia/reperfusion injury in diabetic rats [19]. Therefore, further toxicological tests should be performed. However, it was conclusive that the mortality of the animals was reduced when they were pretreated with TBHQ (Table 1).

Rats in the BDL group had 50% mortality in 48 h, while the TBHQ+BDL group had 20% mortality with a survival rate of 80%. Furthermore, CCl_4_ at 4 g/kg, i.p., is lethal to 100% of rats intoxicated but pretreatment with TBHQ reduces this mortality by 50%. Common bile duct ligation induces liver damage by accumulating bile acids [23]. The obstruction of bile flow induces severe liver damage, so the mortality rate in experimental animals is very high [24]. Furthermore, cholestasis increases mortality and morbidity in patients undergoing major liver surgery because it is associated with increased sepsis and ischemia/reperfusion injury in the liver, accompanied by endothelial damage, inflammation, increased reactive oxygen species and proinflammatory cytokines, and activation of coagulation and fibrinolysis [25]. Therefore, our results suggest that TBHQ may be a suitable candidate to try to reduce complications related to cholestasis. Furthermore, chronic cholestatic liver diseases induce progressive hepatobiliary damage, with subsequent complications such as fibrosis and cirrhosis, ultimately leading to cancer [5]. Clinically, obstruction of the bile duct generates jaundice, choluria, and hepatomegaly, and, biochemically, it increases plasma liver enzyme markers [26,27]. All animals with obstruction of the bile duct (BDL and BDL+TBHQ groups) presented jaundice (as well as choluria results not shown); micrographs of the BDL group showed necrosis and duct proliferation, although hepatomegaly was not evident (Figure 5, Table 2). The BDL+TBHQ group showed a more preserved macroscopic and microscopic morphology (Figure 3 and Figure 6) than the BDL group. These results were consistent with serum biochemical analyses (Figure 6); animals partially but significantly prevented enzymatic increases in the hepatocellular necrosis marker (ALT) and the cholestasis marker (ALP or GGT). These findings suggest for the first time that TBHQ protects against liver injury induced by obstructive cholestasis.

On the other hand, CCl_4_ is a chlorinated hydrocarbon that was anciently used as a degreaser in household cleaning, industrial factories, dry cleaners, and textile laundries. It was also used in fire extinguishers and as a precursor to refrigerants and propellants, but due to its high oxidative toxicity, it has fallen into disuse; however, some industries still use it [28]. Currently, it is used in scientific research with experimental animals, emulating acute or chronic hepatocellular damage in humans, allowing the evaluation of new strategies to prevent or reverse hepatocellular damage [23]. This toxic solvent is metabolized by cytochrome p450, mainly by CYP2E1, producing free radicals such as CCl_3_* and CCl_2_* that affect lipid metabolism by inhibiting its transport out of hepatocytes and, in turn, increasing lipid synthesis, leading to steatosis [28]. In the present study, we observed steatohepatitis in the groups of rats intoxicated with a sublethal dose of CCl_4_, with extensive areas of ballooning hepatocellular degeneration (Figure 4), previously characterized by enlarged hepatocytes with intermediate filaments embedded in clear cytoplasm in combination with lobular inflammation, necrosis, and steatosis [29]; these morphologic alterations were reduced in the CCl_4_+TBHQ group. In macroscopic analyses, it can be seen that the liver weight/body weight ratio (Table 2) of the CCl_4_ and CCl_4_+TBHQ groups increased considerably, presenting statistically significant differences with respect to the Control group. The higher this value is, the larger the livers are compared to the weight of the rat, indicating the possible presence of hepatomegaly, a very common alteration in livers affected by CCl_4_ metabolism [30]. The inflammation process is a defense mechanism with the aim of limiting and eliminating causes of cell damage, as well as facilitating the tissue repair process. Acute and chronic liver damage is caused by acute inflammation, which is self-limited, contrary to prolonged chronic inflammation common in many diseases, such as cirrhosis [31]. Some studies in animal models of liver damage have described that TBHQ is a hepatoprotective agent, such as in the study carried out in male C57bl/6 mice, which showed that TBHQ mitigates acute liver damage induced by CCl_4_ at a dose of 0.1 mL/kg, i.p. (10 mL/kg body weight volume CCl_4_/volume olive oil = 1:99); another work showed that TBHQ exerts anti-inflammatory activity in liver injury ischemia and reperfusion in male Sprague‒Dawley rats [32,33]. In addition, some in vitro studies suggest that TBHQ at high doses (100 and 500 µM) is cytotoxic in human and murine hepatoma cell lines, respectively [13]. Furthermore, a recent review described the beneficial and toxic effects of TBHQ, suggesting that more research on public health and the mechanism of action in different organs and cells should be carefully explored [34]. Therefore, the present work made a comparison of two different types of liver damage, demonstrating for the first time the anticholestatic effect of TBHQ. 

Because TBHQ presents a wide variety of biological effects [10,11,12], it is possible that it has multiple molecular targets in addition to the already known targets; therefore, online platforms were consulted to predict its possible molecular targets (Table 3). Fifty-six proteins were identified, of which two were transcription factors: Nrf2 (Protein ID: NFE2L2) and p65 (Protein ID: RELA). TBHQ has been associated with the activation of the Nrf2 pathway in HepG2 cells [32] and protects against ischemia/reperfusion-induced liver injury through the activation of the Keap1/Nrf2/ARE signaling pathway in rats [33]. Keap1 represses the Nrf2/ARE pathway in two ways: in the cytoplasm, Keap1 recruits Nrf2 into the Cul3/containing E3 ubiquitin ligase complex of Cul3/containing E3, causing its proteasomal degradation; however, Keap1 is able to translocate to the nucleus to dissociate Nrf2 from ARE [35]. Furthermore, the activation of the Nrf2/ARE signaling pathway induced by TBHQ is inhibited by the NF-κB subunit p65 through its interaction with Keap1, which induces the nuclear export of Nrf2 [36,37]. In the present work, the interaction between TBHQ and Keap1 was hypothesized.

Furthermore, in Table 3, other target proteins were identified, such as the nuclear factor-kappa B p105 subunit (Protein ID: NFKB1), which is a suppressor of inflammation [38]; the nuclear receptor ROR gamma liver (Protein ID: RORC), considered a potential therapeutic target in liver fibrosis [39]; xanthine dehydrogenase (ID: XDH), which catalyzes the oxidation of NADH-generating tissue injuries mediated by reactive oxygen species [40]; and the bile acid receptor (Protein ID: NR1H4), whose low expression is related to biliary atresia characterized by fibrous obstruction in childhood liver diseases [41]. These, among other molecules, make up 96.4% of the identified protein targets, for which the effects of TBHQ are unknown. The meticulous analyses in this table open up the possibility of further studies involving these proteins in liver disease.

## 4. Materials and Methods

### 4.1. Animals

A total of 36 male Wistar rats weighing between 200 and 250 g were used in the CCl_4_ model and 40 male Wistar rats weighing 300 to 350 g were used in the bile duct ligation model (BDL). All rats were kept with ad libitum water and a standard diet (LabDiet 5008) [42] and 12 h of light and 12 h of darkness at 24 °C. Experimental procedures in rats were approved by the Research Ethics Committee of the Huasteca Zone Faculty of Professional Studies of the Autonomous University of San Luis Potosí, México, and were carried out based on international terms and guidelines, as well as technical specifications for the production, care, and use of laboratory animals dictated by the official Mexican standard NOM-062-ZOO-1999 [43].

### 4.2. Preparation of TBHQ

To be administered orally, 100 mg of TBHQ (Sigma‒Aldrich, Saint Louis, MO, USA) was dissolved in 100 μL of dimethylsulfoxide (DMSO, Sigma‒Aldrich, Saint Louis, MO, USA) and then diluted with buffered sterile phosphate saline (PBS, pH 7.4), with constant stirring in the dark to a final concentration of 5 mg/mL TBHQ in 1% DMSO and administered immediately after preparation.

### 4.3. Experimental Protocol of the CCl_4_ Model

#### 4.3.1. Administration of TBHQ to Rats Intoxicated with CCl_4_

Pretreatments with TBHQ (40 mg/kg, i.p., every 8 h) [21,22] started three days before CCl_4_ intoxication [19]. 

#### 4.3.2. Mortality Assessment during the Lethal Dose of CCl_4_

**Figure 11:**
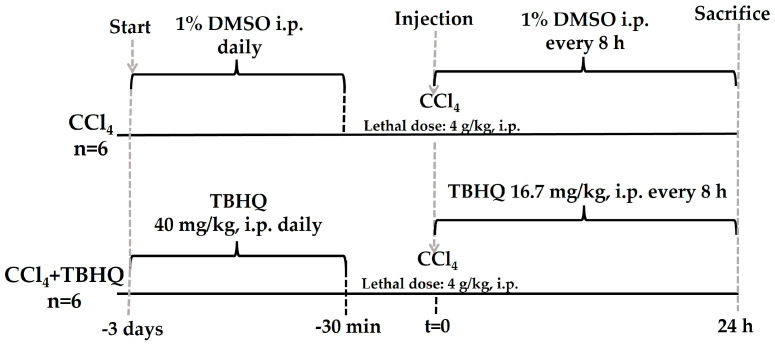
In vivo experimental design to evaluate the ability of TBHQ to prevent rat mortality from a lethal dose of CCl_4_. Twelve rats were divided into two groups for the lethal toxicity assay of CCl_4_ (4 g/kg, i.p.): CCl_4_ (n = 6) and CCl_4_+TBHQ (n = 6). CCl_4_: carbon tetrachloride; TBHQ: *Tert*-butylhydroquinone; DMSO: dimethylsulfoxide (vehicle for TBHQ); i.p.: intraperitoneal route.

Twelve rats were divided into two groups (Figure 11). CCl_4_ group (n = 6): lethal dose of CCl_4_ (4 g/kg, i.p.) [44] and TBHQ+CCl_4_ group (n = 6): TBHQ+CCl_4_ (4 g/kg, i.p.). Mortality was recorded 24 h after CCl_4_ administration.

#### 4.3.3. Hepatoprotective Activity of TBHQ during the Sublethal Dose of CCl_4_

**Figure 12:**
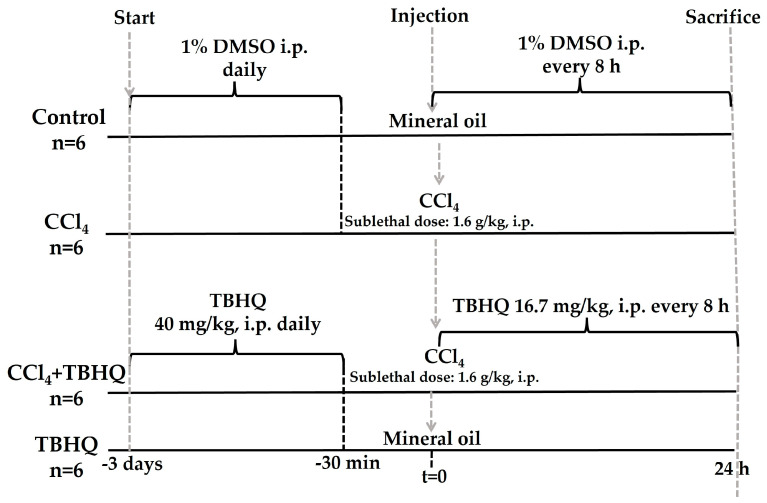
Experimental protocol to evaluate the hepatoprotective capacity of TBHQ in acute liver damage induced by a sublethal dose of CCl_4_. Twenty-four rats were divided into four groups for the sublethal toxicity test of CCl_4_ (1.6 g/kg, i.p.): Control (n = 6), CCl_4_ (n = 6), CCl_4_+TBHQ (n = 6), and TBHQ (n = 6). CCl_4_: carbon tetrachloride; TBHQ: *Tert*-butylhydroquinone; DMSO: dimethyl sulfoxide (vehicle for TBHQ); i.p.: intraperitoneal route.

Twenty-four rats were divided into four groups (Figure 12). Control group (n = 6), CCl_4_ group (n = 6): sublethal dose of 1.6 g/kg, i.p., of CCl_4_ [45,46], TBHQ+CCl_4_ group (n = 6): TBHQ+CCl_4_ (1.6 g/kg, i.p.), and TBHQ group. Mineral oil (CCl_4_ vehicle) was administered i.p. to the Control and TBHQ groups. Survival was recorded after 24 h of CCl_4_ or mineral oil administration.

### 4.4. Experimental Protocol of the BDL Model

**Figure 13:**
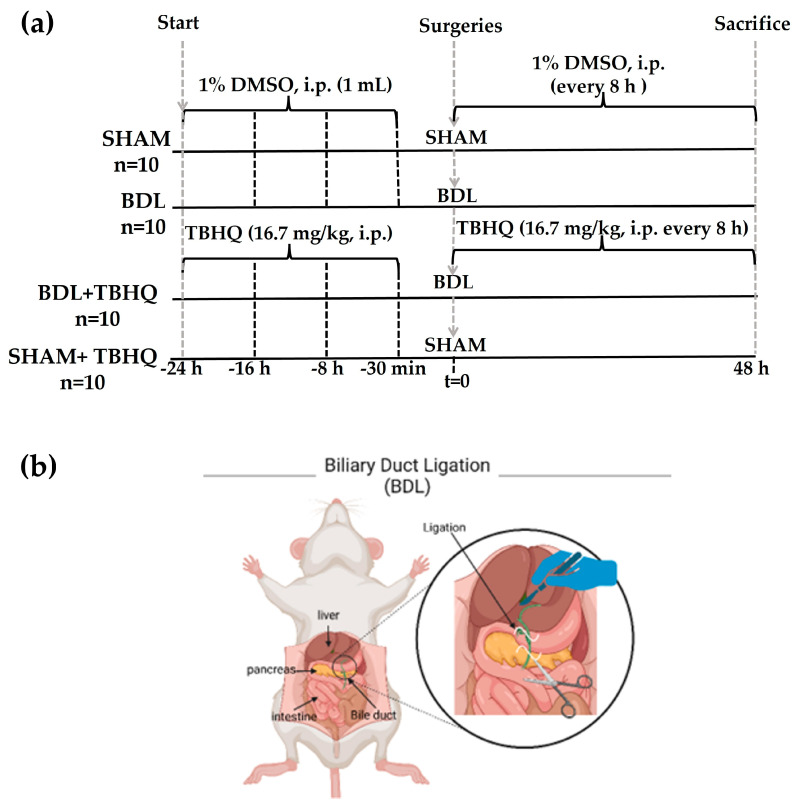
Experimental protocol to evaluate the hepatoprotective effect of TBHQ on acute liver damage induced by extrahepatic cholestasis: (**a**) general protocol and (**b**) schematic representation of the surgical process of BDL (image created from BioRender.com). A total of 40 rats were divided into four groups: SHAM (n = 10), BDL (n = 10), BDL+TBHQ (n = 10), and SHAM+TBHQ (n = 10).

The rats were divided into four groups (Figure 13). BDL group (n = 10): rats were previously administered 1% DMSO (1 mL, i.p., every administration, every 8 h) for a total of four doses before surgery, which consisted of performing a double ligation in the common bile duct, one close to the duodenum and another proximal to the liver; finally, the bile duct was cut off [26,47] and administration of 1% DMSO continued every 8 h until sacrifice. BDL+TBHQ group (n = 10): rats were pretreated 24 h before BDL with three oral administrations of TBHQ (16.7 mg/kg, i.p., every 8 h) and the fourth administration was carried out 30 min before the surgical procedure. After surgery, the administrations continued every 8 h until sacrifice. SHAM group (n = 10): simulated surgery rats were treated with 1% DMSO. SHAM+TBHQ group (n = 10): animals with SHAM surgery and TBHQ administration. Mortality in rats from the acute liver cholestasis model was recorded 48 h after undergoing SHAM or BDL surgical procedures. Finally, all animals were sacrificed 48 h after SHAM or BDL surgeries.

### 4.5. Sacrifice of Experimental Animals

The rats were weighed and immediately anesthetized with an i.p. mixture of xylazine hydrochloride (Sigma‒Aldrich) at 10 mg/kg and ketamine hydrochloride (Sigma‒Aldrich) at 80 mg/kg and immediately sacrificed by cardiac puncture. The blood collected was centrifuged (1000× *g*, 10 min, 4 °C) to obtain serum. Furthermore, the liver was dissected, washed in a cold saline solution, and weighed. Small liver fragments were then obtained and immersed in 4% p-formaldehyde, pH 7.0.

### 4.6. H&E Staining Procedure

Liver tissues fixed for 48 h with 4% p-formaldehyde pH 7.0 were progressively dehydrated with organic solvents as follows (1 h each): 70% ethanol, 80% ethanol, 96% ethanol, absolute ethanol, ethanol-xylene 1:1, xylene (twice), and hot paraffin (twice). Next, tissues were embedded in paraffin, and 4 µm histological sections were prepared on an Ecoshel model 202A microtome and fixed on slides treated with 2% 3-aminopropyltriethoxysilane in acetone [48]. Next, they were subjected to deparaffinization at 60 °C for 24 h. H&E staining was carried out in the following order: xylol 10 min, xylol 1 min, absolute ethanol-xylol (1:1) 1 min, absolute ethanol 1 min, ethanol 96% 1 min, ethanol 80% 1 min, hematoxylin 5 min, wash with tap water 6 min, acid alcohol 0.5% 1 s, distilled water 1 min, ammonia solution 0.05% 5 min, distilled water 1 min, distilled water 1 min, eosin 1 min, 80% ethanol 1 min, 96% ethanol 1 min, absolute ethanol 1 min, absolute ethanol-xylene (1:1) 1 min, and twice with xylene (1 min each).

### 4.7. Photographic Images of H&E Staining

H&E stains were visualized with a Zeiss Axioscope 40/40 FL microscope and analyzed with ImageJ version 1.53e software.

### 4.8. Evaluation of Serum Biochemical Markers of Liver Damage

The enzyme activities of ALT [49], GGT [50], and ALP [51] were evaluated in serum samples. Briefly, plasma ALT enzyme activity was evaluated in duplicate for each test: 250 μL of substrate solution (0.2 M D/L of alanine with 2 M α-ketoglutaric acid) and 50 μL of serum were mixed and incubated at 37 °C for 60 min. Subsequently, 250 μL of the chromogenic reagent (1 mM 2,4-dinitrophenylhydrazine) was added and the sample was further incubated for 15 min at the same temperature. Finally, 1.5 mL of 0.4 N NaOH was added and measured on a spectrophotometer at a wavelength of 515 nm. The enzyme activity of ALP was determined in each sample in duplicate by adding 250 μL of 0.1 M glycine buffer, 1 mM MgCl_2_ with a pH of 10.5, and 250 μL of p-nitrophenylphosphate substrate, mixing and incubating at 37 °C for 5 min. After that time, 50 μL of serum was added to incubate again at 37 °C for 30 min. Finally, NaOH 0.02 N was added and absorbances were measured in a spectrophotometer with a wavelength of 410 nm. The GGT enzymatic activities were carried out in duplicate in each sample with 400 μL of 0.2 M Tris-HCl reagent, 100 μL of MgCl_2_, 100 μL of 0.04 M glycyl-glycine, and 100 μL of 10 mM gamma-glutamyl-p-nitroanilide. Once the solution was prepared, it was incubated for 10 min at 37 °C, after which 200 μL of serum to evaluate was added, and it was incubated again for 30 min at the same temperature of 37 °C. After incubation, 2 mL of 1.5 M acetic acid was added to stop the reaction, and the absorbance was measured in a spectrophotometer at a wavelength of 410 nm. For the three markers of liver damage, blanks were included, and the respective standard curves were performed as suggested by the authors.

### 4.9. Statistical Analysis

Statistical analysis was performed using GraphPad Prism 8.00 software. The results of the biochemical studies were expressed as the mean values ± SE from each experimental group, and comparative analysis was carried out using variance analysis followed by Tukey’s test. Statistical significance was considered at *p* < 0.05.

### 4.10. Identification of Potential Targets for TBHQ Protein in the Liver

Eight online platforms, where protein targets for chemical compounds can be searched or predicted, were consulted to analyze the types of proteins with which TBHQ could interact. The platforms used were ChEMBL [52,53], PharmMapper [54,55,56], Pharos [57], PPB [58], SEA [59], Super-PRED [60], SwissTargetPrediction [61], and TargetNet [62], and they were consulted between 5 and 14 July 2022. On these platforms, the TBHQ molecule was submitted either by drawing it or in mol2 or SMILES formats, with both generated on the ChemInfo website using OpenBabel software [63,64], where the molecule was drawn to obtain them. From each set of data obtained, the protein targets with the highest probability of being the said targets were chosen, according to the platform algorithm and if indicated in the results (PharmMapper, Super-PRED, SwissTargetPrediction, and TargetNet), or all those obtained by the other platforms were further used. Of the protein targets chosen, their protein code was searched in the GeneCards database [65,66] if it was not listed in the results of each platform. With this code, the Human Protein Atlas, a large compendium that integrates results from omics technologies to map human protein expression in cells, tissues, and organs, was consulted [67].

### 4.11. Molecular Docking of TBHQ with Keap1

#### 4.11.1. Keap1/Nrf2 Protein

**Figure 14:**
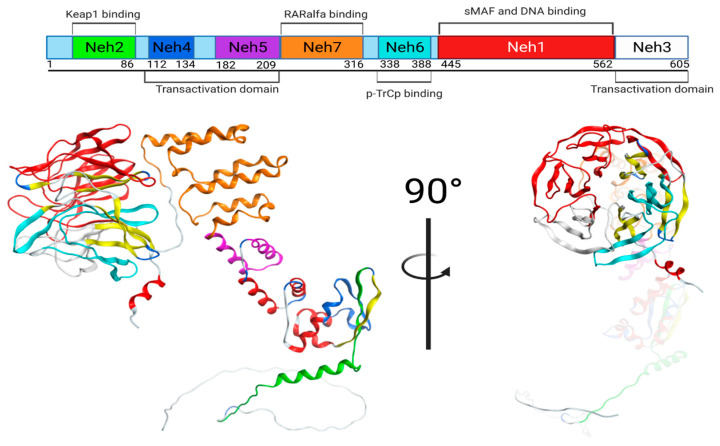
Schematic representation of the domains of Nrf2.

The Nrf2 is a transcription factor with seven domains: Neh 1, Neh 2, Neh 3, Neh 4, Neh 5, Neh 6, and Neh 7 (Table 6 and Figure 14).

#### 4.11.2. Molecular Docking

We analyzed the interaction of TBHQ by looking for molecules that inhibit the interaction between Nrf2 and Keap1, since there are crystallized structures of Keap1 with small molecules in the interaction domain between them that may function as controls for this putative interaction. First, PDB 5CGJ, a Keap1 crystal structure of Keap1, bound to RA839, a small molecule that binds noncovalently to the Keap1–Kelch domain and affects its interaction with Nrf2 (Kd = 6 μM) [68]. This structure has an X-ray diffraction resolution of 3.36 Å, an R-value of 0.226, and an R-value of 0.137. A preliminary analysis identified the amino acids in Keap1 that interact with RA839. The second PDB used was 7C60, which contains the Keap1 crystal in a complex with MEF, which is the metabolite of the Nrf2-activating drug dimethyl fumarate used for the treatment of multiple sclerosis [69]. This structure has an X-ray resolution of 1.95 Å, an R-value of 0.288, and an R-value of 0.214. A preliminary analysis was performed to identify amino acid residues involved in the interaction between Keap1 and MEF. Finally, the interaction between Keap1 and Nrf2 was screened using PDB 7K2M, which has a resolution of 2.02 Å, an R-value of 0.261, and an R-value of 0.237 [70]. Preliminarily, we analyzed which Keap1 residues are involved in this interaction.

Molecular docking analysis was prepared and performed with the best-resolved Keap1 protein from PDB 7C60 using MOE 2022.02 (Chemical Computing Group, Molecular Operating Environment (MOE), 2022.02 Chemical Computing Group ULC, 1010 Sherbrooke St. West, Suite #910, Montreal, QC, Canada, H3A 2R7, 2022). The protein was parameterized using the AMBER 14: EHT forcefield, water molecules were removed, and the structure was minimized. Subsequently, the cocrystallized ligands were removed. By using SiteFinder from MOE, a site was found where some of the already identified residues (shown in Figure 8) and the tested small molecules (RA839, MEF, and TBHQ) could be hosted, and a set of dummy atoms was placed near the site. With this information, that site was used as a receptor for molecular docking. The receptor was programmed to be rigid, and the search algorithm was based on the geometry of Triangle Matcher with London dG as the scoring function to obtain 100 poses. The protocol was followed by a pose refinement using the GBVI/WSA dG scoring function to filter 10 of the most energetically favorable conformations of protein‒ligand complexes [71,72,73,74,75,76,77,78,79].

## 5. Conclusions

Our results suggest for the first time that the administration of TBHQ increases survival in animals with extrahepatic cholestasis or by lethal toxicity of CCl_4_ and propose new protein targets that can be evaluated as possible protection mechanisms for TBHQ against liver disease. In our proof-of-concept model, we found that TBHQ has a tendency to introduce deeper into the protein structure compared to RA839, MEF, and the Nrf2–Kelch domain, which explains its binding energy being greater than MEF but still lower than RA839; nevertheless, the interaction and complementarity of TBHQ in Keap1 could have a major influence on inhibition of the Keap1–Nrf2–Keap1 forming trimer Keap1–Nrf2–Keap1, which could cause an increase in Nrf2 concentration into the cytoplasm to be translocated to the nucleus, then activating the expression of antioxidant proteins responsible for the hepatoprotective effect. Even when TBHQ could not interact directly with the residues involved in the formation of the Keap1–Nrf2 complex, based on the idea that even a small change in the geometry of the binding site could cause a large change in protein conformation, the deeper introduction of our ligand into Keap1 could cause an impediment to Nrf2 binding and then increase its concentration, causing the abovementioned effect.

## Figures and Tables

**Table 1:** Mortality and survival of CCl_4_ and BDL experimental models.

Liver Damage Model	Mortality		
	(n)	%	Survival %
**Lethal dose of CCl_4_**			
CCl_4_ (4 g/kg, i.p.)	6/6	100	0
CCl_4_ (4 g/kg, i.p.) + TBHQ	3/6	50	50
**Sublethal dose of CCl_4_**			
CCl_4_ (1.6 g/kg, i.p.)	0/6	0	100
CCl_4_ (1.6 g/kg, i.p.) + TBHQ	0/6	0	100
**BDL experimental model**			
BDL	5/10	50	50
BDL+TBHQ	5/10	20	80

**Table 2:** Body and liver weight of the rats in hepatotoxicity model with sublethal dose of CCl_4_ and BDL model.

Group	Body Weight (g)	Liver Weight (g)	Liver/Body Weight
**Control**	348.3 ± 4.70	11.5 ± 0.65	0.033 ± 0.00169
**CCl_4_**	350.8 ± 12.98	16.2 ± 0.67 ^a^	0.046 ± 0.00194 ^ac^
**CCl_4_+TBHQ**	302.9 ± 8.26 ^ab^	15.21 ± 0.76 ^a^	0.050 ± 0.00178 ^ac^
**TBHQ**	370.3 ± 9.53	13.92 ± 0.37	0.038 ± 0.00010
**SHAM**	411.6 ± 3.32	16.23 ± 0.061	0.039 ± 0.00179
**BDL**	373.38 ± 16.55	15.36 ± 0.76	0.040 ± 0.00323
**BDL+TBHQ**	366.9 ± 11.62	14.48 ± 0.13	0.039 ± 0.00143
**SHAM+TBHQ**	335.93 ± 12.43	14.6 ± 0.49	0.043 ± 0.00056

Results were expressed as the mean ± SE. Statistically significant differences, *p* < 0.05: ^a^ significantly different from the Control group; ^b^ significantly different from the CCl_4_ group; ^c^ significantly different from the SHAM+TBHQ group.

**Table 3:** Putative protein targets for TBHQ in liver found in different databases.

Target Protein Name	Protein ID	Target Location and/or Function (Human Protein Atlas)	Target Search Platform
Nuclear factor erythroid 2-related factor	NFE2L2 (Nrf2)	Antioxidant Liver—Metabolism	P
Nuclear factor-kappa B p105 subunit	NFKB1	NF-KB subunit	SP
Transcription factor p65	RELA	NF-KB subunit	TN
Acetyl-CoA carboxylase 2	ACACB	Cell type enhanced—Hepatocytes	SP
Estrogen receptor	ESR1	Cell type enhanced—Hepatocytes	PM, S, TN
Endoplasmic reticulum-associated amyloid beta-peptide-binding protein	HSD17B10	Cell type enhanced—Hepatocytes	P, SP
Dihydropteridine reductase	QDPR	Cell type enhanced—Hepatocytes	S
Nuclear receptor ROR gamma	RORC	Cell type enhanced—Hepatocytes	P
Excitatory amino acid transporter 3	SLC1A1	Cell type enhanced—Hepatocytes	SP
Transthyretin	TTR	Cell type enriched—Hepatocytes	PM, SP
Tyrosine-protein kinase receptor RET	NRTN	Cell type enhanced—Hepatocytes	SP
Oxysterols receptor LXR-alpha	NR1H3	Cell type enhanced—Hepatocytes	TN
Glyceraldehyde-3-phosphate dehydrogenase, liver *	GAPDH	Tissue enhanced—Liver	ST
Hydroxysteroid 17-beta-dehydrogenase 3	HSD17B3	Tissue enhanced—Liver	TN
Pyruvate dehydrogenase kinase 4	PDK4	Cell type enriched—Liver–Hepatocytes	S
Aryl hydrocarbon receptor	AHR	Liver—Metabolism	TN
Transient receptor potential cation channel subfamily V member 1	TRPV1	Liver—Metabolism	TN
Aldo-keto reductase family 1 member C3	AKR1C3	Liver—Lipid metabolism	PM
Monoamine oxidase A	MAOA	Liver—Lipid metabolism	SP, TN
Androgen receptor	AR	Liver—Metabolism Cell type enhanced—Hepatocytes	PM, TN
Coagulation factor X	F10	Liver—Metabolism Cell type enhanced—Hepatocytes	PM
Monoamine oxidase B	MAOB	Liver—Metabolism Cell type enhanced—Hepatocytes	TN
Bile acid receptor	NR1H4	Liver—Metabolism Cell type enhanced—Hepatocytes	S
Cholinergic receptor nicotinic alpha 4 subunit (neuronal acetylcholine receptor)	CHRNA4	Liver—Metabolism Cell type enhanced—Hepatocytes	SP
Cocaine esterase	CES2	Liver—Lipid metabolism Cell type enhanced—Hepatocytes	TN
Nuclear receptor subfamily 1 group I member 2 (pregnane X receptor)	NR1I2	Liver—Lipid metabolism Cell type enhanced—Hepatocytes	SP, PM
Estrogen sulfotransferase	SULT1E1	Liver—Lipid metabolism Cell type enhanced—Hepatocytes	PM
Xanthine dehydrogenase	XDH	Liver—Lipid metabolism Cell type enhanced—Hepatocytes	Ph, SP, TN
Serum albumin	ALB	Tissue enriched—Liver Liver—Hemostasis Cell type enriched—Hepatocytes	PM, ST
Complement factor B	CFB	Tissue enriched—Liver Liver—Hemostasis Cell type enhanced—Hepatocytes	PM
Prothrombin	F2	Liver—Hemostasis Cell type enriched—Hepatocytes	PM
Hydroxysteroid 11-beta dehydrogenase 1	HSD11B1	Liver—Hemostasis and lipid metabolism Cell type enriched—Hepatocytes	SP, TN
Butyrylcholinesterase	BCHE	Liver—Hemostasis and lipid metabolism Cell type enhanced—Hepatocytes	Ch
Carbonic anhydrase 5A, mitochondrial	CA5A	Liver—Hemostasis and lipid metabolism Cell type enriched—Hepatocytes	TN
Liver carboxylesterase 1 *	CES1	Liver—Hemostasis and lipid metabolism Cell type enriched—Hepatocytes	TN
Cytochrome P450 1A2	CYP1A2	Liver—Hemostasis and lipid metabolism Cell type enriched—Hepatocytes	TN
Cytochrome P450 2C9	CYP2C9	Liver—Hemostasis and lipid metabolism Cell type enriched—Hepatocytes	P, TN
IgG receptor FcRn large subunit p51	FCGRT	Liver—Lipid metabolism Cell type enhanced—Kupffer cells	SP
Arachidonate 5-lipoxygenase	ALOX5	Cell type enhanced—Kupffer cells	SP, ST, TN
C-C chemokine receptor type 2	CCR2	Cell type enhanced—Kupffer cells	SP
Tyrosine-protein kinase FGR	FGR	Cell type enhanced—Kupffer cells	SP
Formyl peptide receptor 1	FPR1	Cell type enhanced—Kupffer cells	SP
Lipoxin A4 receptor	FPR2	Cell type enhanced—Kupffer cells	SP
G-protein coupled bile acid receptor 1	GPBAR1	Cell type enhanced—Kupffer cells	SP
Tyrosine-protein kinase SYK	SYK	Cell type enhanced—Kupffer cells	PM
Hydroxysteroid17-beta-dehydrogenase 2	HSD17B2	Liver—ER transport pathway Cell type enhanced—Hepatocytes	S
Endoplasmic reticulum aminopeptidase 1	ERAP1	Liver—ER transport pathway	SP
Tissue factor pathway inhibitor	TFPI	Tissue enhanced liver Liver—ER transport pathway	SP
Tyrosine-protein kinase YES	YES1	Liver—ER transport pathway	SP

All those with the highest probability and that, according to the curated data from the Human Protein Atlas, had a liver-related tissue and/or cellular localization, and were specifically indicated (*) to be a liver protein. Ch—ChEMBL; PM—PharmMapper; Ph—Pharos; P—PPB; S—SEA; SP—Super-PRED; ST—SwissTargetPrediction; TN—TargetNet; ER—Endoplasmic reticulum.

**Table 4:** Potential binding site identified by SiteFinder. In bold, the amino acid residues that also participate in the interactions between Keap1 and the small molecules RA839 and MEF are highlighted.

Site	Size	Hyd	Side	Residues
Approximately the highest propensity for ligand binding	192 alpha spheres (with the size of a carbon atom sphere)	42 hydrophobic contact atoms in the receptor	64 side-chain contact atoms in the receptor	TYR334 SER363 GLY364 LEU365 ALA366 GLY367 CYS368 ASN382 ASN414 **ARG415** ILE416 GLY417 VAL418 GLY419 VAL420 GLY462 VAL463 GLY464 VAL465 ALA466 VAL467 **SER508** GLY509 ALA510 GLY511 VAL512 CYS513 VAL514 **TYR525 GLN530 SER555 ALA556** LEU557 GLY558 ILE559 THR560 VAL561 **TYR572 PHE577 SER602** GLY603 VAL604 GLY605 VAL606 ALA607 VAL608

**Table 5:** Interaction energies resulting from the molecular docking analysis.

Ligand	Nef2/Nrf2	MEF	RA839	TBHQ
Energy (kcal/mol)	−10.4673	−5.2186	−6.9461	−5.5491

**Table 6:** Nrf2 domains.

Domain	Name	Residues/Amino Acids
Neh 1	sMAF and DNA binding	435–562
Neh 2	Keap1 binding	50–86
Neh 3	Transactivation domain	563–605
Neh 4	Transactivation domain	112–134
Neh 5	Transactivation domain	182–209
Neh 6	p-TrCp binding	338–388
Neh 7	RARalfa binding	210–316

## Data Availability

The data presented in this study are available on request from the corresponding author J.R.M.-P.
